# Context-driven discovery of gene cassettes in mobile integrons using a computational grammar

**DOI:** 10.1186/1471-2105-10-281

**Published:** 2009-09-08

**Authors:** Guy Tsafnat, Enrico Coiera, Sally R Partridge, Jaron Schaeffer, Jon R Iredell

**Affiliations:** 1 Centre for Health Informatics, Univ. of New South Wales, Sydney, NSW 2052, Australia; 2 Centre for Infectious Diseases and Microbiology, Univ. of Sydney, Westmead Hospital, Sydney, NSW 2145, Australia

## Abstract

**Background:**

Gene discovery algorithms typically examine sequence data for low level patterns. A novel method to computationally discover higher order DNA structures is presented, using a context sensitive grammar. The algorithm was applied to the discovery of gene cassettes associated with integrons. The discovery and annotation of antibiotic resistance genes in such cassettes is essential for effective monitoring of antibiotic resistance patterns and formulation of public health antibiotic prescription policies.

**Results:**

We discovered two new putative gene cassettes using the method, from 276 integron features and 978 GenBank sequences. The system achieved *κ *= 0.972 annotation agreement with an expert gold standard of 300 sequences. In rediscovery experiments, we deleted 789,196 cassette instances over 2030 experiments and correctly relabelled 85.6% (*α *≥ 95%, *E *≤ 1%, mean sensitivity = 0.86, specificity = 1, F-score = 0.93), with no false positives.

Error analysis demonstrated that for 72,338 missed deletions, two adjacent deleted cassettes were labeled as a single cassette, increasing performance to 94.8% (mean sensitivity = 0.92, specificity = 1, F-score = 0.96).

**Conclusion:**

Using grammars we were able to represent heuristic background knowledge about large and complex structures in DNA. Importantly, we were also able to use the context embedded in the model to discover new putative antibiotic resistance gene cassettes. The method is complementary to existing automatic annotation systems which operate at the sequence level.

## Background

Computational methods for discovering DNA functions typically seek similarities with sequences of known genes [1-4]. Numerous methods used to assist in the annotation of nucleotide and peptide sequences rely on a "most-similar known feature" principle [5-9] cross-referencing to information from public databases (*e.g*. GenBank [10], protein sequence repositories [11], and ontologies [12]).

Annotating or discovering more complex patterns in sequences which arise from the assembly of multiple discrete sequence units requires a different approach. For example, the genes involved in conferring bacterial resistance to antibiotics are found in structures that are repetitive, mosaic and follow patterns governed by molecular processes (*e.g*. transposition), and are subjected to evolutionary selection pressures [13]. The difficulty in finding such patterns is that they are functionally similar but have quite different base-pair sequences. Integrons, for example, were initially identified from repeated manual observations of a similar sequence pattern (restriction enzyme digestion sites) flanking a variety of antibiotic resistance genes [14].

Understanding the biological "rules" that govern pattern assembly should allow for the automated analysis of complex structures that can systematically reveal new motifs. Formal computational grammars have found some application to complex sequence analysis in the past [15,16]. For example, gene promoters have quite variable base-pair sequences but knowledge of the molecular mechanisms they participate in allows a general definition of promoter structure to be formalised [17]. Similarly, unrelated self-annealing regions of RNA can be analysed to indicate interesting secondary structures [18].

The method presented here uses a context-sensitive deterministic grammar to parse higher order DNA structures, *i.e*. assemblies of genetic features. Such features may be known DNA sequence units identified via homology-based annotations or compositions of such units into more complex structures. By analogy with human language, such a grammar focuses on the ways in which words assemble into phrases and sentences, rather than current sequence methods which focus on how letters assemble into words.

We evaluate this approach on the task of discovering bacterial gene cassettes associated with class 1, 2 and 3 integrons [19]. A typical gene cassette consists of little more than a single promoter-less gene, (often conferring antibiotic resistance) and a DNA recombination site (*attC*). Many genes that confer resistance to most classes of antibiotics (including disinfectants) are found in gene cassettes, along with many open reading frames of as yet unknown function [20]. These gene cassettes thus form a large gene pool of major importance in antibiotic resistance management.

An integron is a gene cassette capture and expression element that is characterized by another DNA recombination site (*attI*), an integrase gene (*intI*) and a promoter (Pc). Interaction between *attI *and *attC*, catalysed by the IntI protein, results in the insertion of the corresponding gene cassette into the integron. An arbitrary number of cassettes may be inserted into the same integron, always in the same orientation, to create an array. Class 1, 2 and 3 integrons are important as they contribute to the movement of antibiotic resistance genes between DNA molecules, *e.g*. into mobile plasmids that can then travel between bacteria, including different species, facilitating spread of resistance.

Class 1 integrons are the most clinically important and usually include two conserved segments (known as the 5'-CS and 3'-CS) flanking a variable region consisting of the inserted gene cassettes. In some examples the 3'-CS is not present and a *tni *transposition region instead marks the end of the cassette array. Equivalent conserved flanking regions are also present in class 2 integrons and the few class 3 integrons that have been identified to date. These easily recognisable flanking regions allow amplification of the variable cassette arrays by the polymerase chain reaction (PCR) with subsequent DNA sequencing to identify the gene cassettes present. This has resulted in the submission of large numbers of cassette arrays to the GenBank database. Unfortunately, many of the annotations are incomplete. Most notably, in many gene cassettes only the gene is annotated, without the flanking regions, *attC *site, or array context. Manually re-annotating cassette arrays and compiling results to look for patterns that might help to predict spread of multi-antibiotic resistance is both difficult and time-consuming. Annotation algorithms for cassette arrays need to be flexible enough to accept a variable number of cassettes and truncated features from errors in the cassette insertion process, random deletions, insertions of other mobile elements and artifacts from the amplification and/or sequencing process.

The task of annotating integrons may be supported by several classes of computational tools:

*Metagenomic Annotators *[5,21] use open reading frame (ORF) and gene prediction tools (primarily Glimmer [22]) and gene and protein function knowledge repositories such as UniProt [11], the Kyoto Encyclopedia of Genes and Genomes [23] and the Gene Ontology [12] to label genes and assign functions to them. While such tools do not label integrons automatically, one can use them to find *intI *genes to anchor searches for *attI *and *attC *sites.

*Integron Annotation Support Tools and Databases *provide reference repositories for integrons and their components. The databases are searchable using keywords or BLAST queries. Compilation of the databases is a manual or semi-automatic process. INTEGRALL [24] depends on user submissions for database updates. ARDB [25] is a manually curated reference for antibiotic resistance genes including those found in integrons. ACID [26] include a set of tools that include models of *attC*, *attI *and integron structure to accurately annotate the integrons down to the level of *attC *and *attI *site subparts. XXR [27] is a tool that predicts *attC *sites and ORFs using regular expressions and other heuristics and can help curate and verify gene cassettes in such databases. Because these databases are curated by human experts and provided as reference tools, their accuracy has not been reported in the literature.

*Automatic Integron Structure Model Annotators *are tools that automatically label integrons and their parts (*attI *sites, *intI *genes, gene cassettes, 3'-CS, 5'-CS *etc*.), and when possible, assign higher order annotations such as gene products and function. In this paper we present what we believe to be the first implementation in this class of annotators. The mosaic nature of integrons means that using existing similarity-based algorithms (*e.g*. BLAST [28], FASTA [29]) to annotate an integron would require comparison with every possible cassette combination. However, the number of different cassettes [30], and therefore the number of possible combinations, is too high for this to be feasible.

Other approaches to annotating larger-than-gene regions in DNA rely on comparative genomics to find commonalities among sequences from different species but none is used for the annotation of integrons or other mobile genetic elements. It is yet unclear how they would handle the wide variations observed in integrons [30]. Mauve [31] is a global alignment tool that identifies regions common to multiple species and aligns their assemblies. It is primarily used in phylogeny of eukaryotes. MetaMine [32] is an automatic annotation tool for multi-gene clusters in bacterial chromosomal DNA. Given a target gene, MetaMine examines DNA sequences from multiple species to find other genes that frequently co-occur with it. It then uses meta-genomics to hypothesize a function fulfilled by the cluster.

The datasets and annotations used in this study as well as the software and source code are available for download for free for non-commercial purposes from the authors'. web site: http://www2.chi.unsw.edu.au/attacca/

## Methods

### Sequence Annotation

We manually assembled a test set of 276 features associated with integrons from the literature into a feature database (FDB). Each feature comprised of a unique identifier, a name, a type (*e.g*. gene cassette), a model sequence and a minimum identity criterion. Minimum identity criteria were assigned to each feature and varied between 95% and 100%. Features representing gene cassettes included the gene and the *attC *parts and, following common practice, were named after the gene they carry. A complete list of features and a description of how they were selected is in [20].

A complementary test set of 978 unique sequences from the GenBank CoreNucleotides database that contained at least one of these integron features, into a sequence database (SDB). GenBank entries with the word "vector" or "synthetic construct" in their organism field, a few cassette array sequences with long stretches of Ns representing un-sequenced regions (e.g. DQ915900-DQ915939) as well as RefSeq entries were excluded.

To ensure we had a uniform annotation of SDB for our experiments, FDB was used to annotate the sequences in three stages. 1. BLAST [28] was used to tag all occurrences of the features in SDB. Matches that met a minimum percent identity and length criteria were kept. The minimum length criterion for all matches was 25 bases, as a lower limit introduced spurious annotations of short fragments irrelevant to the annotation. 2. Manual annotation was used to tag 257 features in SDB not recognised by BLAST. In most cases (85.6%), less than 25 bases of the feature were sequenced. The manual annotations were based on the sequence annotation in GenBank or on information published in the literature that accompanied the sequence (*e.g*. the PCR primers used). 3. Any remaining annotation gaps in SDB were then used as queries in FDB, using BLAST. This allowed occurrences of truncated features to be identified. Features truncated at both ends were not considered. 4. The parser may re-label annotations assigned using BLAST given new context information [see Additional File 1].

Annotations using the FDB were stored in a relational database for analysis by the grammar. This step allows other annotations sources, including GenBank annotations, to be used independently or in conjunction with our annotation system. The exact position of feature annotations does not affect the structure annotation as long as their order is not changed.

### Grammar

**Figure 1:**
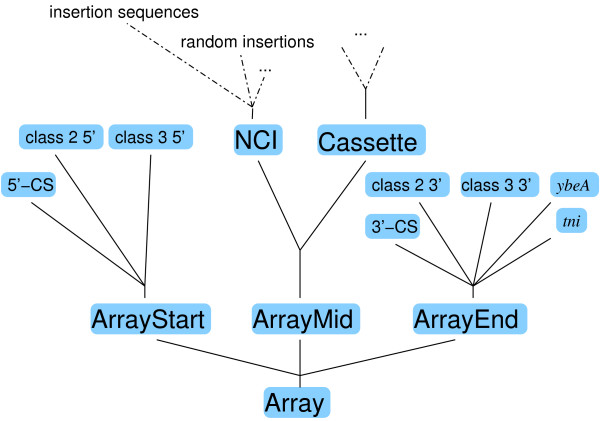
**The cassette array grammar**. The start of the array is marked by an integron-class specific 5' flanking sequence or *attI *and its end with the corresponding 3' flanking sequence, *tni *or *ybeA*. The middle consists of a number of cassettes and non-cassette insertions (NCI) such as insertion sequences.

The first and third authors manually developed a 21-rule grammar to describe the structure of cassette arrays [see Additional File 1] based on known molecular mechanisms for integron assembly (Figure 1) published in the literature [19]. The grammar is a direct formal representation of the model proposed by Hall and Collis [19] with some modifications, for example, to accommodate non-cassette insertions.

**Figure 2:**
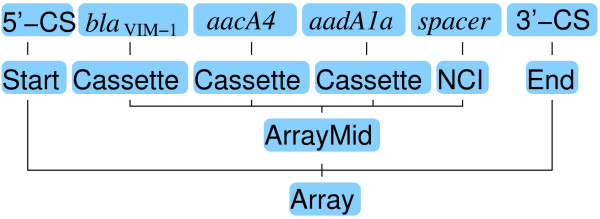
**Visual representation of a tree resulting from a parse of one array sequence containing three gene cassettes and a non-cassette insertion**.

Grammar rules have the form *C*_1 _*X C*_2 _::= *C*_1 _*y*_1 _... *y*_*n*_*C*_2_, which implies that *X*, when occurring in the context {*C*_1_, *C*_2_} is a structure that consists of a sequence of tokens *y*_1 _... *y*_*n*_. For example, a rule to identify an array as a structure consisting of a sequence of three lower level features might be:

The rules were used by a context-sensitive deterministic parser which reads token sequences from left to right, and builds its parse tree bottom-up, from the leaf nodes (lowest level features) of the grammar, and which allowed incomplete parses (Figure 2). We found that using a context-sensitive grammar (CSG) notation in which multiple symbols are allowed on the left hand side of the rule operator (::=) allowed us to map biological knowledge to grammar rules more easily. While a CSG was not strictly necessary, it required fewer and simpler grammar rules than a context-free grammar would have. The relatively small number of rules (21) and the relatively short number of annotations in each sequence (none had more than 30 annotations) made the added computational complexity of parsing with a CSG was insignificant.

Structures can occur in opposite directions even in the same sequence (*e.g*. GenBank accession AY509004). Structure direction is ensured by the parser which checks that component features are oriented appropriately or match specified rules. For example, a sequences such as  will not be ambiguous and thus incorrectly recognised as an array structure because the ArrayEnd is not oriented from left to right.

A special class of rules, called *discovery rules*, that can predict unannotated cassettes occurring in specific contexts, were derived from the 21-rule grammar. For example, for the Array rule above, the following discovery rule is generated:

where *λ *represents a gap in annotation *i.e*. an unannotated sequence fragment.

This discovery rule is used to hypothesise that a gap in annotation occurring between the start and end of an array is a cassette. In this way, discovery rules allow the parser to find only specific contexts in which gaps should be tested as new cassettes. This reduces the number of gaps identified to only those that can create an array from two incomplete array parts separated by the gap. Seven such cassette discovery rules were developed and added to the basic 21-rule grammar [see Additional File 1]. An argument was added to *λ *tokens so that sequences too short to be considered cassettes or long gaps between different cassette arrays were not hypothesised to be cassettes. The minimum (300 bp) and maximum (1860 bp) were calculated based upon the lengths of the longest non-cassette insertion, features that occur in arrays but are not cassettes and don't interrupt the integrity of the array, and the longest cassettes in FDB.

If a feature is identified using a discovery rule, and the feature is not present in FDB, then we can hypothesise that this is a new example of the feature class, and add it to FDB as a potential new discovery. The feature is then available for subsequent sequence analyses, allowing the feature lexicon to grow adaptively as the system encounters new features.

### Rediscovery Experiments

To evaluate the ability of our method to discover previously unseen gene cassettes, we randomly excluded known cassettes from our FDB, and tested the method's capacity to rediscover them from instances in SDB. Ten omission proportions were tested from 5% to 50% at 5% increments. In each experiment a random subset of cassettes was omitted from the FDB. Whenever features were rediscovered from SDB they were incorporated into the FDB and the annotation was repeated until no new discoveries were made. Of the 214 gene cassettes in FDB, ten were not present in the SDB, two only appear without context (*e.g*. GenBank accession AB074436) and eight never appear in an array context (*e.g*. Z86002). In such cases, context based discovery is impossible and so these features were not tested in the experiments.

In this experiment gaps that were labelled as cassettes by discovery rules are counted as true positives if the gaps corresponded in position to cassettes in the gold standard. All other gaps identified as cassettes were considered false positives. Gaps that corresponded to cassettes in the gold standard but which were not identified as such were counted as false negatives, and all remaining features where the annotation agreed with the gold standard were counted as true negatives.

### Statistical Analysis

Sample size calculations for each omission proportion were calculated from a pilot study of 50 experiments (Table 1). The number of experiments required for each class was calculated for a confidence interval of *α *≥ 0.95, and error margin of *E ≤ *1%. Normal distribution for the pilot study was verified using the Anderson-Darling test [33]. Due to the 100% upper boundary limit for rediscovery, the Kolmogorov-Smirnov test for a truncated normal distribution (*x *∈ [0, 1]) with the estimated mean and SD (0.797 ± 0.124) was used. The KS test could not reject the null-hypothesis that the distribution of rediscoveries at the 5% omission proportion was normal.

**Table 1:** Sample SD based on a pilot study with *n *= 50 experiments in 5 omission proportions for a desired confidence *α *≥ 95% and a desired Error *E *≤ 1%.

Omission Proportion	SD	A*^2^	Required Experiments	KS test *p*-value
5%	0.140	1.09	749	0.068
10%	0.096	0.565	352	
15%	0.081	0.733	252	
20%	0.070	0.505	187	
25%	0.054	0.300	113	
30%	0.052	0.419	103	
35%	0.044	0.716	75	
40%	0.044	0.360	74	
45%	0.041	0.438	66	
50%	0.039	0.150	59	

The Anderson-Darling values, adjusted for sample size (A*^2^) greater than 0.752 rejects the null-hypothesis that sample is normally distributed. For the 5% omission proportion, a truncated normal distribution was tested using the KS test which gave a *p*-value > 5%.

## Results and Discussion

### New Cassette Discoveries

We let the discovery system annotate 978 unique sequences from GenBank with the 276 features associated with integrons. We then ran the context sensitive parser with both structure and discovery rules on the annotated sequences. The system detected two putative gene cassettes. We manually verified that they were indeed gene cassettes that had not previously been reported [20]:

The first was found in EF522838 (3564..4127), submitted to GenBank in 2007, and encoded a protein 81% identical to QacE, which is associated with resistance to antiseptics [34]; the cassette was designated *qacK *. The other was found in DQ993182 (64..540), submitted to GenBank in 2006, and encoded a protein related to DfrB proteins (85% identical to DfrB2), which confer resistance to trimethoprim [35]; the cassette was designated *dfrB7 *.

### Gold Standard Agreement

A sample of 300 sequences (30.6%) containing 1585 integron features was selected randomly from our test set. Three microbiologists were given the name and location of each feature and were asked to independently classify them into one of five categories: cassette, array start marker, array end marker, a non-cassette part of an array, or a non-cassette feature occurring outside an array. A gold standard annotation was created based upon the majority expert classification for these features.

The automated system also attempted the same classification task. Each expert completed the task independently. Agreement between the three experts, measured using Fleiss's kappa [36], was *κ *= 0.975. Agreement between the experts and the grammar was *κ *= 0.972. A value of *κ *≥ 0.8 is considered very strong agreement [37].

The gold standard annotation consisted of 4280 features including cassettes, conserved sequences and non-cassette insertions covering 24.7 Mb. The annotation also included 872 gaps covering 2.3 Mb.

### Cassette Rediscovery

In a further trial, we automatically annotated the GenBank sequences with all of the features in the feature database (FDB) and conducted 2030 experimental runs, deleting a randomly selected subset of cassette annotations, to test the ability of the method to rediscover the deleted cassettes based upon their local context alone, and without recourse to cassette sequence information. We included in the experiments 194 cassettes that appeared within an array context in the GenBank sequences, and excluded 20 cassettes which had one or more context elements missing from the sequences, as these are not discoverable with our method.

**Figure 3:**
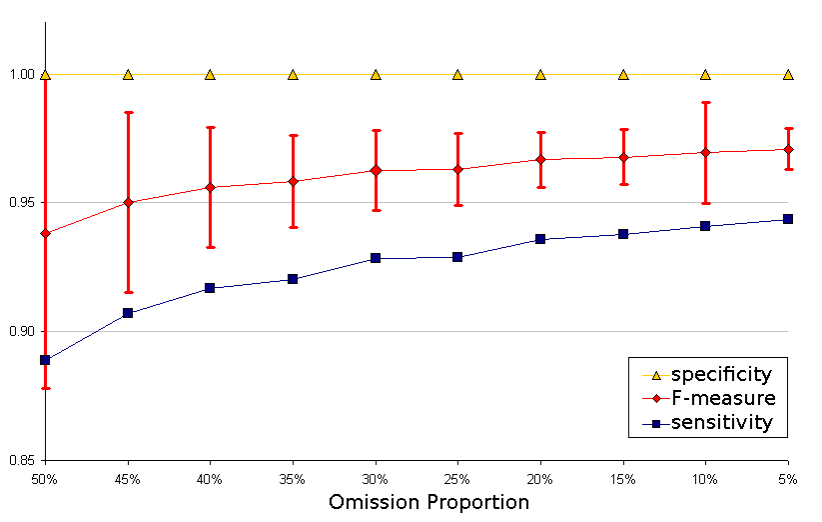
**Sensitivity, specificity and F_1_-measure from comparison of annotations made with rediscovered cassettes and the gold standard annotation**. Error bars indicate ± 1SD in F_1_-measure.

In total we deleted 789,196 cassette instances in the 2030 experimental runs. We correctly relabelled 675,940 cassette deletion instances as a cassette, yielding an average of 85.6% (*α *> 95%, *E *< 1%) of unannotated cassettes that were successfully rediscovered. When the annotations were compared to the expert gold standard annotation, the algorithm had a mean specificity of 1 and specificity 0.86, achieved an overall F-score [38] of 0.93 (Figure 3) and had no false positives. There was little variation in discovery rates with different sample sizes of randomly omitted cassettes, but confidence intervals in the discovery rate narrowed with increase in the number of deleted cassettes, as expected (Figure 3).

On average, we found 25,343 true positives, 4,246 false negatives, 362,536 true negatives and zero false positives per million base-pairs.

If the 10 excluded gene cassettes not possessing flanking contexts are considered, then the discovery system was able to correctly annotate (*i.e*. True Positive Rate = 1) 83.3% of the sequences. All of the 194 theoretically discoverable cassettes were rediscovered at least once.

To the best of our knowledge, there are currently no available tools to automatically annotate integrons that these results can be compared against. We identified 349 unique cassette arrays each occurring between 1 and 50 times (median = 1), hence repeated BLAST searches for the known arrays would be ineffective. For the same reason, a gene clustering approach such as MetaMine [32] would not identify the majority of arrays as they are not found often enough to be recognized as a cluster. Manual annotation of novel gene cassettes involves BLAST searching for the known start and end markers, predicting genes between them, for example using ORF Finder [39], and then identifying the *attC *sites that flank those genes by manual inspection [20,40]. Alternatively, gene cassettes can be found by repeated BLAST searches with the 132 known gene cassettes. However, this method is limited to finding homologs only. Both methods are labour intensive, time consuming and error prone.

### Error Analysis

In 113,256 of the cassette deletions, the algorithm missed the presence of a cassette in a sequence, resulting in an apparent false negative rate of 14.4%. Detailed examination of these false negatives suggest that in each instance two cassettes occurred side-by-side in the GenBank sequence, resulting in the following error cases:

#### Type I

Two adjacent cassettes are identified as a single cassette. This error occurred in 72,338 of the deletion instances and if these were to be reclassified as true positives (as the cassettes were correctly identified but incompletely resolved) then the overall algorithm performance increases by 9.1% to produce an overall 94.8% discovery rate, a specificity of 0.92 and F-score of 0.96. Use of some additional sequence information should eliminate this type of error entirely *e.g*. searching for *attC *sites [27].

#### Type II

Two adjacent cassettes are missed because the combined sequence length of both cassettes exceeded 1860 bp, the maximum length currently used by our algorithm to identify candidate cassettes. This error occurred for 19,896 (2.5%) of the deletions. The upper limit was determined based on the length of the longest cassette occurring in FDB. Setting a higher limit would begin to introduce false positive errors, usually annotation of long regions between fragments from separate integrons. Our current algorithm setting is thus optimised to minimise false positives at the expense of generating Type II errors, and different settings are possible depending upon specific task requirements.

#### Type III

Two cassettes are missed because no other complete features such as flanking regions were present on the sequence, which typically contained only the cassettes themselves, and so no context was available to trigger cassette discovery. This error occurred in 21,022 deletions (2.6%). Our grammar requires at least one annotated feature to be present on a sequence, *i.e*. one of a pair of consecutive cassettes must be annotated so it can act as a bounding context for the other. Common genetic approaches truncate array start and end markers in DNA sequences and our method would not detect a cassette or array without these contextual elements as identifiable bounding features. This may also be overcome by adding a specific search for features such as *attC *sites.

The results show that a grammar approach to sequence analysis can identify genetic features like gene cassettes, hypothesising their functional role by examining the context within which they occur, and without recourse to sequence analysis. Although some low level grammars have been used in the past, based upon sequence patterns, to our knowledge this is the first demonstration of the existence and use of a higher order feature grammar. It is important to note that the grammar method is complementary to existing automatic annotation systems which operate at the sequence level. In particular, our error analysis identified specific circumstances in the integron recognition task where our grammar could not resolve whether cassettes existed or not, because of incomplete information about context in the sequence fragments we analysed, but where some lower level sequence information such as the existence of an *attC *site may be able to resolve feature identity or role.

Consequently, our results reinforce the view that the "language of DNA" supports formal grammatical contracts of a higher order, which can decode genetic function at a higher level of abstraction than base sequence patterns, and even gene patterns. In the domain of integrons, the grammar is demonstrated to unambiguously identify between 84-95% of cassettes, with an upper bound of about 97.4% set by Type III errors. The method is likely to be easily generalisable to any other higher-order DNA structures that can be expressed using rules in a grammar. The evolutionary and biological basis for the development and role of such grammars is likely to be a fruitful level of enquiry for some time to come.

### Future Work

The work reported here represents a proof of concept for using heuristic grammars to model high-order structures in DNA. In this work we labelled cassette using a single consistent nomeclature system, but there remain disagreements in the biology community about the best nomenclature to use. In a future implementation multiple nomeclature systems would be supported. This may have implications for some features as their defining sequences may have slight differences between systems. While the parser is not restricted to a particular annotation source or nomeclature system, the algorithms to integrate annotations from a variety of sources, detect and resolve conflicting annotations and mapping between semantic types of features are yet to be defined.

Extension of the grammar to cover a comprehensive set of structures responsible for antibiotic resistance transmission will require new features in the FDB and additional rules in the grammar. Other mechanisms, such as transposons and composite transposons, are present in fewer sequences in GenBank than gene cassettes and validation of the grammar would require a different approach. The validation method might consist of comparisons of the method's annotations with a random guessing baseline, and/or a database of "synthetic" sequences. In our experiments, ten of the unique 204 (5.2%) cassettes found in SDB were not discoverable because they lacked sufficient context. Further evaluation is required to test if less context is sufficient to accurately predict the presence of cassettes so that this rate can be reduced.

The discovery rules presented here all rely on contextual information to be present on both sides of the discovered feature. In almost 1000 naturally occurring sequences, this resulted in no false positives and hence a specificity of 1.0. Rules using only single sided contexts can be used to increase the sensitivity of the algorithm, but at a cost to specificity.

## Conclusion

We have shown that using a grammar to represent heuristic background knowledge allows feature identification based on local context alone, without recourse to feature sequence data. We were able to automatically annotate bacterial mobile elements and achieve a high level of agreement with a panel of experts. Significantly, the grammar also facilitated discovery of putative new antibiotic resistance gene cassettes through inspection of the contexts at which gaps in the annotation occur. Up to 97.5% of genetic features in our domain appear to be discoverable by context-driven discovery using a grammar.

## Authors' contributions

GT designed and implemented the annotation and discovery algorithms, implemented the grammar and drafted the manuscript. EC participated in system design, guided the experimental design and helped draft the manuscript. SRP constructed the feature database, provided the biological background to the grammar and contributed the biological background to the manuscript. JS conducted the pilot study and rediscovery experiments. JRI participated in the design of the system. All authors read and approved the final manuscript.

## Supplementary Material

Additional file 1 **Cassette Array Grammar**. The grammar used in this work. Click here for file
