# Supplementary material for: Context-driven discovery of gene cassettes in mobile integrons using a computational grammar
Source: BMC Bioinformatics. 2009 Sep 8;10:281. doi: 10.1186/1471-2105-10-281 (PMC3087341; doi:10.1186/1471-2105-10-281)
Supplement: Additional file 1 — Cassette Array Grammar. The grammar used in this work. [file 1471-2105-10-281-S1.pdf]

Cassette Array Grammar

The following are the grammar rules used to annotate cassette arrays and discover cassettes. Terminal tokens matched by name are in quotes, otherwise token symbols refer to semantic type. Directions on all left hand side tokens have to agree except for λ tokens that have no directionality. If direction is not noted, either direction can be used as long as the direction for all tokens is the same. Square brackets denote a definite clause range for the length of a λ token.

1 Structure Rules

`Cassette`	::=	"*aadA2*" | ... 214 cassettes ...
`NonCassetteInsertion`	::=	"*attI1* `L-spacer`" | "`IS1`" | ... 38 features ...
`ArrayEnd`	::=	"`3′-CS`" | "`class 2 3′`" | "`class 3 3′`" | "*ybeA*"
`ArrayStart`	::=	"`5′-CS`" | "`class 2 5′`" | "`class 3 5′`"
`ArrayMid`	::=	`Cassette`
`ArrayEnd`	::=	`ArrayEnd` `ArrayEnd`
`ArrayStart`	::=	`ArrayStart` `ArrayStart`
`ArrayMid`	::=	`ArrayMid` `ArrayMid`
$\overrightarrow{\texttt{Array}}$	::=	$\overrightarrow{\texttt{Array}}$ $\overrightarrow{\texttt{ArrayEnd}}$
$\overleftarrow{\texttt{Array}}$	::=	$\overleftarrow{\texttt{Array}}$ $\overleftarrow{\texttt{ArrayStart}}$
$\overrightarrow{\texttt{Array}}$	::=	$\overrightarrow{\texttt{ArrayStart}}$ $\overrightarrow{\texttt{ArrayEnd}}$
$\overleftarrow{\texttt{Array}}$	::=	$\overleftarrow{\texttt{ArrayEnd}}$ $\overleftarrow{\texttt{ArrayStart}}$
$\overrightarrow{\texttt{ArrayStart}}$ $\overrightarrow{\texttt{ArrayMid}}$	::=	$\overrightarrow{\texttt{Array}}$ $\overrightarrow{\texttt{ArrayMid}}$
`ArrayMid` `ArrayMid`	::=	`NonCassetteInsertion` `ArrayMid`
`ArrayMid` `ArrayMid`	::=	`ArrayMid` `NonCassetteInsertion`
$\overleftarrow{\texttt{ArrayEnd}}$ $\overleftarrow{\texttt{ArrayMid}}$	::=	$\overleftarrow{\text{“}tni\text{”}}$ $\overleftarrow{\texttt{ArrayMid}}$
$\overrightarrow{\texttt{ArrayEnd}}$ $\overrightarrow{\texttt{ArrayMid}}$	::=	$\overrightarrow{\texttt{ArrayMid}}$ $\overrightarrow{\text{“}tni\text{”}}$
$\overrightarrow{\texttt{Array}}$	::=	$\overrightarrow{\texttt{ArrayStart}}$ $\overrightarrow{\texttt{ArrayMid}}$ $\overrightarrow{\texttt{ArrayEnd}}$
$\overleftarrow{\texttt{Array}}$	::=	$\overleftarrow{\texttt{ArrayEnd}}$ $\overleftarrow{\texttt{ArrayMid}}$ $\overleftarrow{\texttt{ArrayStart}}$
$\overrightarrow{\texttt{ArrayMid}}$ λ $\overrightarrow{\texttt{ArrayEnd}}$	::=	$\overrightarrow{\texttt{ArrayMid}}$ λ $\overrightarrow{\text{“}tni\text{”}}$
$\overleftarrow{\texttt{ArrayEnd}}$ λ $\overleftarrow{\texttt{ArrayMid}}$	::=	$\overleftarrow{\text{“}tni\text{”}}$ λ $\overleftarrow{\texttt{ArrayMid}}$

The cassette carrying the *qacE* gene is identical in its first 390 bp to a conserved sequence typically marking the end of an array in a class 1 integron ("`3′-CS`"). If a truncated version of the cassette with less than 390 bp is found, the cassette is incorrectly labelled as a truncated "`3′-CS`" by the annotator. The rule $\overrightarrow{\texttt{ArrayStart}}\overrightarrow{\texttt{ArrayMid}}$ ::= $\overrightarrow{\texttt{Array}}\overrightarrow{\texttt{ArrayMid}}$ corrects the annotation be relabelling the truncated "`3′-CS`" to a truncated *qacE* gene cassette so that the cassette array is then correctly annotated.

2 Discovery Rules

$$
\begin{array}{rcl}
\overrightarrow{\texttt{ArrayStart}}\ \overrightarrow{\texttt{Cassette}}\ \overrightarrow{\texttt{ArrayEnd}} & ::= & \overrightarrow{\texttt{ArrayStart}}\ \lambda\texttt{[300-1860]}\ \overrightarrow{\texttt{ArrayEnd}} \\
\overleftarrow{\texttt{ArrayStart}}\ \overleftarrow{\texttt{Cassette}}\ \overleftarrow{\texttt{ArrayEnd}} & ::= & \overleftarrow{\texttt{ArrayStart}}\ \lambda\texttt{[300-1860]}\ \overleftarrow{\texttt{ArrayEnd}} \\
\overrightarrow{\texttt{ArrayStart}}\ \overrightarrow{\texttt{Cassette}}\ \overrightarrow{\texttt{ArrayMid}} & ::= & \overrightarrow{\texttt{ArrayStart}}\ \lambda\texttt{[300-1860]}\ \overrightarrow{\texttt{ArrayMid}} \\
\overleftarrow{\texttt{ArrayMid}}\ \overleftarrow{\texttt{Cassette}}\ \overleftarrow{\texttt{ArrayStart}} & ::= & \overleftarrow{\texttt{ArrayMid}}\ \lambda\texttt{[300-1860]}\ \overleftarrow{\texttt{ArrayStart}} \\
\overrightarrow{\texttt{ArrayMid}}\ \overrightarrow{\texttt{Cassette}}\ \overrightarrow{\texttt{ArrayEnd}} & ::= & \overrightarrow{\texttt{ArrayMid}}\ \lambda\texttt{[300-1860]}\ \overrightarrow{\texttt{ArrayEnd}} \\
\overleftarrow{\texttt{ArrayEnd}}\ \overleftarrow{\texttt{Cassette}}\ \overleftarrow{\texttt{ArrayMid}} & ::= & \overleftarrow{\texttt{ArrayEnd}}\ \lambda\texttt{[300-1860]}\ \overleftarrow{\texttt{ArrayMid}} \\
\texttt{ArrayMid}\ \texttt{Cassette}\ \texttt{ArrayMid} & ::= & \texttt{ArrayMid}\ \lambda\texttt{[300-1860]}\ \texttt{ArrayMid}
\end{array}
$$
